# Staphylococcus aureus ATP Synthase Promotes Biofilm Persistence by Influencing Innate Immunity

**DOI:** 10.1128/mBio.01581-20

**Published:** 2020-09-08

**Authors:** Megan E. Bosch, Blake P. Bertrand, Cortney E. Heim, Abdulelah A. Alqarzaee, Sujata S. Chaudhari, Amy L. Aldrich, Paul D. Fey, Vinai C. Thomas, Tammy Kielian

**Affiliations:** a Department of Pathology and Microbiology, University of Nebraska Medical Center, Omaha, Nebraska, USA; New York University School of Medicine

**Keywords:** biofilm, cytokines, macrophages, myeloid-derived suppressor cell

## Abstract

Staphylococcus aureus is a major cause of prosthetic joint infection (PJI), which is characterized by biofilm formation. S. aureus biofilm skews the host immune response toward an anti-inflammatory profile by the increased recruitment of myeloid-derived suppressor cells (MDSCs) that attenuate macrophage proinflammatory activity, leading to chronic infection. A screen of the Nebraska Transposon Mutant Library identified several hits in the ATP synthase operon that elicited a heightened inflammatory response in macrophages and MDSCs, including *atpA*, which encodes the alpha subunit of ATP synthase. An *atpA* transposon mutant (Δ*atpA*) had altered growth kinetics under both planktonic and biofilm conditions, along with a diffuse biofilm architecture that was permissive for leukocyte infiltration, as observed by confocal laser scanning microscopy. Coculture of MDSCs and macrophages with Δ*atpA* biofilm elicited significant increases in the proinflammatory cytokines interleukin 12p70 (IL-12p70), tumor necrosis factor alpha (TNF-α), and IL-6. This was attributed to increased leukocyte survival resulting from less toxin and protease production by Δ*atpA* biofilm as determined by liquid chromatography with tandem mass spectrometry (LC-MS/MS). The enhanced inflammatory response elicited by Δ*atpA* biofilm was cell lysis-dependent since it was negated by polyanethole sodium sulfanate treatment or deletion of the major autolysin, Atl. In a mouse model of PJI, Δ*atpA*-infected mice had decreased MDSCs concomitant with increased monocyte/macrophage infiltrates and proinflammatory cytokine production, which resulted in biofilm clearance. These studies identify S. aureus ATP synthase as an important factor in influencing the immune response during biofilm-associated infection and bacterial persistence.

## Abstract (precis)

Medical device-associated biofilm infections are a therapeutic challenge based on their antibiotic tolerance and ability to evade immune-mediated clearance. The virulence determinants responsible for bacterial biofilm to induce a maladaptive immune response remain largely unknown. This study identified a critical role for S. aureus ATP synthase in influencing the host immune response to biofilm infection. An S. aureus ATP synthase alpha subunit mutant (Δ*atpA*) elicited heightened proinflammatory cytokine production by leukocytes *in vitro* and *in vivo*, which coincided with improved biofilm clearance in a mouse model of prosthetic joint infection. The ability of S. aureus Δ*atpA* to augment host proinflammatory responses was cell lysis-dependent, as inhibition of bacterial lysis by polyanethole sodium sulfanate or a Δ*atpA*Δ*atl* biofilm did not elicit heightened cytokine production. These studies reveal a critical role for AtpA in shaping the host immune response to S. aureus biofilm.

## INTRODUCTION

Staphylococcus aureus is an opportunistic pathogen that expresses a wide array of virulence determinants to evade host immune responses (1–4). S. aureus can asymptomatically colonize several sites of the human body, most often in the anterior nares, where approximately 30% of individuals are persistent S. aureus carriers and up to 60% of the population may be intermittent carriers (5, 6). A patient’s carrier status is a risk factor for postsurgical S. aureus infection (7, 8). This is particularly relevant for arthroplasty procedures, as S. aureus is a frequent cause of prosthetic joint infection (PJI) with methicillin-resistant S. aureus (MRSA) strains responsible for up to half of these infections (9, 10). As a result, patients who are S. aureus carriers are subjected to decolonization protocols prior to arthroplasty as a standard of care at many medical institutions (11, 12). Not only are PJIs often associated with bacteria that harbor genes that encode antibiotic resistance, but they are also typified by biofilm formation, which affords antibiotic tolerance and dampens host proinflammatory immune responses (1, 13, 14).

Biofilms are communities of bacteria encased by a self-produced matrix consisting of proteins, carbohydrates, and extracellular DNA (eDNA) (15, 16). The extracellular matrix provides structure to the biofilm and also allows for nutrient distribution and the exchange of substrates (17). Additionally, there is metabolic diversity within the biofilm, which provides rapid adaptation to stressors and antibiotic tolerance (16). Our laboratory has previously shown in a mouse model of PJI that S. aureus biofilm can actively suppress proinflammatory responses by the preferential recruitment of myeloid-derived suppressor cells (MDSCs) and anti-inflammatory monocytes/macrophages (MΦs) to the site of infection (14, 18). These MDSCs produce interleukin 10 (IL-10) to create an immunosuppressive environment that allows for biofilm persistence (19, 20). Importantly, MDSC infiltrates are also more pronounced in tissues from patients with PJI than with aseptic loosening, reinforcing the findings in the mouse PJI model (21, 22).

A screen of the Nebraska Transposon Mutant Library (NTML) (23) was conducted to identify mutations that elicited a heightened proinflammatory response from MΦs and MDSCs during coculture with mature S. aureus biofilm. Significant hits occurred in genes within the ATP synthase operon, specifically in *atpA*, *atpD*, and *atpG*. These genes encode the alpha, beta, and gamma subunits of the ATP synthase catalytic core, respectively. ATP synthase is a central metabolic enzyme that is driven by the proton motive force generated by the respiratory chain, and it functions to synthesize ATP (24, 25). With regard to S. aureus, a recent study identified that *atpG* was required for virulence in a mouse model of skin and soft tissue infection (SSTI) (26). This was attributed, in part, to a failure in intracellular acidification, which is required for the optimal activity of fermentative enzymes that generate energy in the face of respiration defects (26). However, the role of S. aureus ATP synthase in influencing biofilm development and subsequent effects on host immunity has not yet been explored. In this report, we show that *atpA* was essential for biofilm persistence in a mouse model of PJI. Disruption of *atpA* reduced toxin and protease production, which resulted in a heightened proinflammatory response due to enhanced leukocyte survival.

## RESULTS

### ATP synthase plays a critical role in dictating biofilm growth and structure.

**FIG 1:**
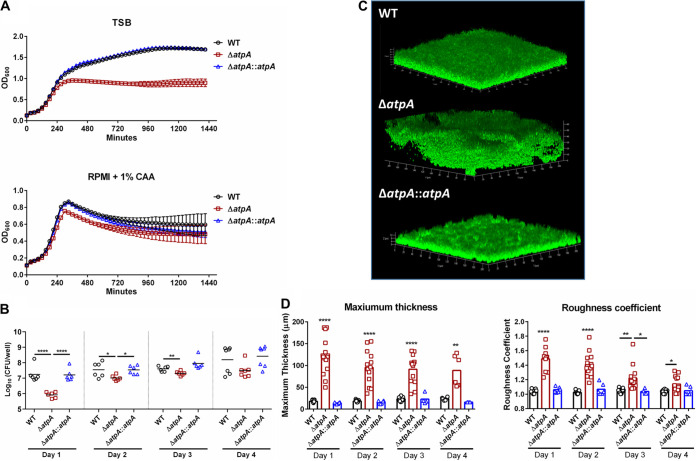
S. aureus Δ*atpA* biofilm displays early growth defects and altered structure. (A and B) The growth of S. aureus WT, Δ*atpA*, and Δ*atpA*::*atpA* was characterized by OD_600_ in tryptic soy broth (TSB) or RPMI-1640 supplemented with 1% Casamino Acids (CAA; mean ± SD of one representative experiment; *n* = 6 biological replicates) (A) and CFU of *in vitro* biofilm at various stages of development (mean combined from 2 independent experiments; *n* = 6 biological replicates) (B). (C) Representative three-dimensional (3D) images of 4-day-old biofilm acquired using confocal laser scanning microscopy. (D) Maximum thickness and roughness coefficient measurements were calculated by Comstat 2 analysis (mean combined from 1 to 4 independent experiments; *n* = 3 to 15 biological replicates). Significant differences are denoted by asterisks (*, *P < *0.05, **, *P < *0.01, and ****, *P < *0.0001; one-way ANOVA with Tukey’s multiple-comparison test).

To characterize the role of S. aureus ATP synthase in influencing MDSC and MΦ activation, we focused on *atpA* since this gene is upstream of *atpD* and *atpG* in the operon and, as such, was also inactivated in the NTML *atpA* mutant. These subunits compose the catalytic core of ATP synthase; therefore, disruption of these genes renders the enzyme nonfunctional. Bacterial ATP synthase is critical for energy production, homeostasis, and maintaining the proton motive force (24). Therefore, we first characterized the growth kinetics of Δ*atpA*, which was assessed in tryptic soy broth (TSB) and RPMI-1640 with 1% Casamino Acids (CAA) under both planktonic and biofilm growth conditions. RPMI-1640 is a standard base medium for eukaryotic cells and was utilized throughout this study for biofilm-leukocyte coculture experiments since it better models the mammalian tissue milieu compared to TSB. Δ*atpA* displayed a postexponential-phase growth defect in both medium formulations compared to wild type (WT) (Fig. 1A). Biofilm burden was reduced in Δ*atpA* during the first 3 days of growth but then reached titers similar to WT biofilm (Fig. 1B). Biofilm architecture was notably different in Δ*atpA*, with an increased maximum thickness and roughness coefficient compared to WT (Fig. 1C to D). All the Δ*atpA* phenotypes were complementable (Fig. 1).

### S. aureus ATP synthase attenuates MDSC and MΦ inflammatory responses to biofilm.

**FIG 2:**
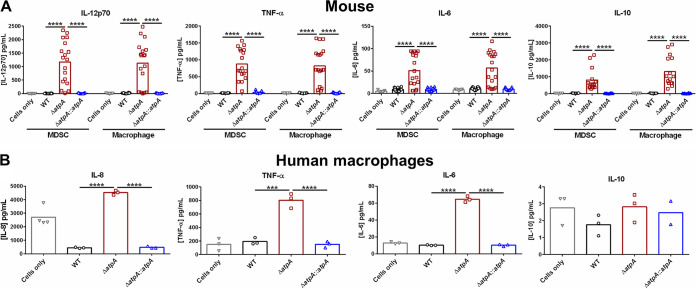
AtpA is critical for attenuating leukocyte cytokine production in response to S. aureus biofilm. S. aureus 4-day-old biofilms were cocultured with 5 × 10^4^ mouse bone marrow-derived MDSCs or macrophages (A) or human monocyte-derived macrophages (B) for 2 h, whereupon cytokine production was quantified using a mouse or human cytometric bead array inflammation kit. Results represent the mean combined from 4 independent experiments (*n* = 7 to 21 biological replicates) (A) and mean of 1 experiment (*n* = 3 biological replicates) (B) repeated with monocytes from 3 different donors. Significant differences are denoted by asterisks (***, *P < *0.001, and ****, *P < *0.0001; one-way ANOVA with Tukey’s multiple-comparison test).

Previous studies from our laboratory have demonstrated that S. aureus biofilm skews leukocytes to an anti-inflammatory state, which promotes bacterial persistence (18, 19, 27). To determine if S. aureus ATP synthase-dependent pathways play a role in this process, primary bone marrow-derived MDSCs and MΦs were cocultured with Δ*atpA* biofilm to quantify cytokine production. MDSCs and MΦs exposed to Δ*atpA* biofilm produced significantly higher levels of the proinflammatory cytokines IL-12p70, tumor necrosis factor alpha (TNF-α), and IL-6 than WT biofilm (Fig. 2A). Although the anti-inflammatory cytokine IL-10 was also significantly elevated in response to Δ*atpA* (Fig. 2A), collectively, the increases in IL-12p70, TNF-α, and IL-6 suggest a proinflammatory bias in response to Δ*atpA* biofilm. These findings were replicated in human monocyte-derived MΦs, where TNF-α, IL-6, and IL-8 production was significantly enhanced in response to Δ*atpA* compared to WT biofilm, whereas IL-10 release was minimal and not affected (Fig. 2B). The increased cytokine production elicited by Δ*atpA* biofilm in mouse and human leukocytes was complementable (Fig. 2). Heightened proinflammatory cytokine release was also elicited by Δ*atpD* and Δ*atpG* biofilm (Fig. S1), highlighting the importance of ATP synthase in influencing leukocyte activation. Additionally, Δ*atpA* was more susceptible to killing by mouse MΦs (Fig. S2), demonstrating that functional ATP synthase renders S. aureus more resistant to MΦ bactericidal activity.

10.1128/mBio.01581-20.1 FIG S1 S. aureus ATP synthase is critical for inhibiting leukocyte cytokine production in response to biofilm. S. aureus *atp* mutants were obtained from the Nebraska Transposon Mutant Library. Bone marrow-derived MDSCs or macrophages were cocultured with 4-day-old biofilm for 2 h, whereupon supernatants were analyzed using a mouse cytometric bead array inflammation kit. Results represent the mean of 1 to 2 independent experiments (*n* = 6 to 9 biological replicates). Significant differences are denoted by asterisks (*, *P < *0.05, **, *P < *0.01, and ****, *P < *0.0001; one-way ANOVA with Dunnett’s multiple-comparison test with WT control). Download FIG S1, TIF file, 0.3 MB. Copyright © 2020 Bosch et al. 2020 Bosch et al. This content is distributed under the terms of the Creative Commons Attribution 4.0 International license.

10.1128/mBio.01581-20.2 FIG S2 S. aureus AtpA influences intracellular survival in macrophages. Macrophages were exposed to S. aureus WT or Δ*atpA* at a multiplicity of infection (MOI) of 1:1, 5:1, or 10:1 (bacteria:macrophage) for 1 h to allow for phagocytic uptake. Next, macrophages were treated with high-dose gentamicin (100 μg/ml) for 1 h to kill residual extracellular bacteria and subsequently incubated in low-dose gentamicin (1 μg/ml) for 1.5 h or 24 h, whereupon macrophages were lysed and intracellular bacteria quantified. Results represent the mean combined from 3 independent experiments (*n* = 9 biological replicates). Significant differences are denoted by asterisks (*, *P < *0.05, **, *P < *0.01, and ****, *P < *0.0001; Student’s *t*-test). Download FIG S2, TIF file, 0.1 MB. Copyright © 2020 Bosch et al. 2020 Bosch et al. This content is distributed under the terms of the Creative Commons Attribution 4.0 International license.

**FIG 3:**
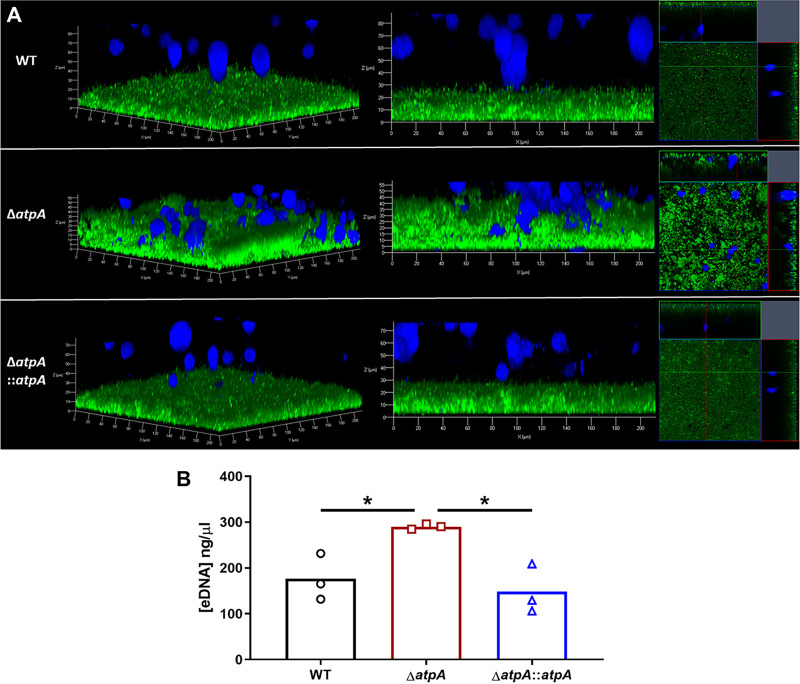
S. aureus AtpA prevents macrophage biofilm invasion and regulates eDNA release. (A) Bone marrow-derived macrophages were stained with CellTracker deep red (pseudocolored blue) and cocultured for 2 h with 6-day-old S. aureus biofilm transduced with a GFP reporter plasmid and imaged by confocal laser scanning microscopy. Representative 3D (left) and side view (middle) z-stack images, as well as orthogonal views (right), are shown from two independent experiments, each with one biological replicate and 5 to 6 images per replicate. (B) S. aureus biofilms were grown in 6-well plates, whereupon eDNA was quantified at day 6 by quantitative PCR. Results represent the mean from one experiment (*n* = 3 biological replicates). Significant differences are denoted by asterisks (*, *P < *0.05; one-way ANOVA with Tukey’s multiple-comparison test).

**FIG 4:**
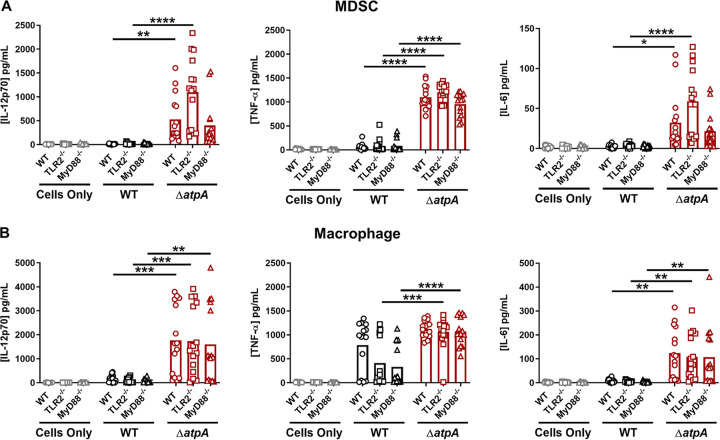
Enhanced proinflammatory mediator production elicited by S. aureus Δ*atpA* biofilm is TLR2- and MyD88-independent. WT, MyD88^−/−^, and TLR2^−/−^ bone marrow-derived MDSCs (A) and macrophages (B) were cocultured with 4-day-old WT or Δ*atpA* biofilm for 2 h, whereupon supernatants were analyzed using a mouse cytometric bead array inflammation kit. Results represent the mean combined from 3 independent experiments (*n* = 15 biological replicates). Significant differences are denoted by asterisks (*, *P < *0.05, **, *P < *0.01, ***, *P < *0.001, and ****, *P < *0.0001, one-way ANOVA with Tukey’s multiple-comparison test).

**FIG 5:**
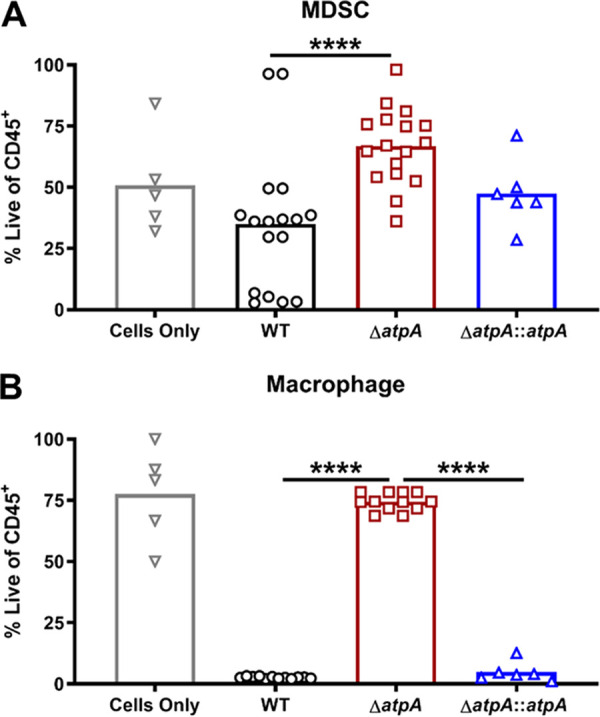
S. aureus AtpA dictates leukocyte survival during biofilm coculture. Bone marrow-derived MDSCs (A) or macrophages (B) were cocultured with 4-day-old biofilm for 2 h, whereupon cell viability was accessed by flow cytometry using a live/dead stain. Results are presented as the percentage of live CD45-positive (CD45^+^) leukocytes and represent the mean combined from 3 independent experiments (*n* = 5 to 17 biological replicates). Significant differences are denoted by asterisks (****, *P < *0.0001; one-way ANOVA with Tukey’s multiple-comparison test).

We next determined if the diffuse structure of Δ*atpA* biofilm (Fig. 1C and D) altered MΦ interactions since previous studies demonstrated that MΦs are unable to invade a WT S. aureus biofilm (28, 29). Δ*atpA* biofilm had more MΦs contacting the biofilm surface as visualized by confocal laser scanning microscopy, whereas MΦs were excluded from WT biofilm (Fig. 3A). The diffuse structure of Δ*atpA* biofilm could make pathogen-associated molecular patterns (PAMPs), such as lipoteichoic acid (LTA), peptidoglycan (PGN), and eDNA, more accessible to invading leukocytes to account for their heightened cytokine production. This possibility was further supported by the finding that eDNA concentrations were significantly increased in Δ*atpA* biofilm (Fig. 3B). S. aureus LTA and PGN are recognized by Toll-like receptor 2 (TLR2), and eDNA engages TLR9, with both TLRs signaling through myeloid differentiation factor 88 (MyD88) (30, 31). To assess the role of PAMPs in potentiating the inflammatory response to Δ*atpA* biofilm, cocultures were performed with TLR2^−/−^ or MyD88^−/−^ MDSCs and MΦs. The response to Δ*atpA* biofilm was equivalent for WT, TLR2^−/−^, and MyD88^−/−^ MDSCs and MΦs, indicating that the heightened cytokine response to Δ*atpA* biofilm was MyD88- and TLR2-independent (Fig. 4). MyD88^−/−^ MDSCs and MΦs were unresponsive to TLR2 (Pam3CSK4 and PGN) and TLR9 (CpG DNA) agonists, confirming defects in TLR signaling (Fig. S3). However, leukocyte viability during the biofilm coculture period revealed increased MDSC and MΦ survival with Δ*atpA* compared to WT biofilm (Fig. 5). Therefore, enhanced cytokine production by leukocytes cocultured with Δ*atpA* biofilm likely results, in part, from an increased number of viable cells being able to sustain cytokine production and not from improved recognition of biofilm antigens.

10.1128/mBio.01581-20.3 FIG S3 MyD88-deficient cells are nonresponsive to Toll-like receptor (TLR) agonists. WT and MyD88^−/−^ bone marrow-derived MDSCs and macrophages were treated with TLR2 (peptidoglycan [PGN], 10 μg/ml; Pam3CysSerLys4 [Pam3CSK4], 100 ng/ml) or TLR9 (CpG oxynucleotide [ODN], 5 μM) agonists for 18 h, whereupon the conditioned medium was analyzed using a mouse cytometric bead array inflammation kit. Results represent 8 biological replicates from one experiment. Significant differences are denoted by asterisks (****, *P < *0.0001; Student’s *t*-test with Holm-Sidak correction). IL-12p70 and IL-10 levels were below the limit of detection and are not shown. Download FIG S3, TIF file, 0.1 MB. Copyright © 2020 Bosch et al. 2020 Bosch et al. This content is distributed under the terms of the Creative Commons Attribution 4.0 International license.

To identify proteins that may contribute to the increased survival of MDSCs and MΦs in response to Δ*atpA* biofilm, liquid chromatography with tandem mass spectrometry (LC-MS/MS) was performed. This analysis revealed a significant reduction in many virulence factors and toxins in Δ*atpA* biofilm supernatants, including serine proteases, α-hemolysin, and leukocidin-like proteins that are known to induce leukocyte death (2) (Table 1; Fig. S4; Data Set S1), supporting the observations of more viable MDSCs and MΦs in Δ*atpA* biofilm cocultures and the lack of hemolysis by Δ*atpA* on blood agar (data not shown). LC-MS/MS also confirmed decreased levels of ATP synthase subunits as well as select metabolic enzymes in Δ*atpA* biofilm extracts (Table 2; Fig. S4B; Data Set S2).

**TABLE 1:** Virulence factors are significantly reduced in supernatants from *ΔatpA* biofilm

Protein	Gene	Log_2_ difference (Δ*atpA*/WT)^*a*^
Serine protease SplC	*splC*	−4.34
Serine protease SplE	*splE*	−3.94
Serine protease SplF	*splF*	−3.89
Serine protease SplB	*splB*	−3.85
Uncharacterized leukocidin-like protein 2	SAB1876c	−2.63
Lipase 1	*lip1*	−2.50
Zinc metalloproteinase aureolysin	*aur*	−2.45
Alpha-hemolysin	*hly*	−2.38
Lipase 2	*lip2*	−2.36
Lysozyme-like protein 7	*lys-7*	−2.02
UPF0173 metal-dependent hydrolase SAUSA300_1653	SAUSA300_1653	−1.92
1-phosphatidylinositol phosphodiesterase	*plc*	−1.75
Uncharacterized leukocidin-like protein 1	SAOUHSC_02241	−1.53
Glutamyl-tRNA(Gln) amidotransferase subunit A	*gatA*	−1.43
Uncharacterized lipoprotein SAOUHSC_02650	SAOUHSC_02650	−1.38
Staphopain A	*sspP*	−1.37
Aspartate carbamoyltransferase	*pyrB*	−1.37
50S ribosomal protein L16	*rplP*	−1.36
Staphylokinase	*sak*	−1.31
Elastin-binding protein EbpS	*ebpS*	−1.30
33-kDa chaperonin	*hslO*	−1.20
50S ribosomal protein L17	*rplQ*	−1.10
Clumping factor A	*clfA*	−1.10
Serine protease SplA	*splA*	−1.03

a All proteins were significantly different; *P *< 0.05.

**TABLE 2:** Metabolic protein expression is reduced in *ΔatpA* biofilm extracts

Protein	Gene	Log_2_ difference (Δ*atpA*/WT)^*a*^
ATP synthase subunit alpha	*atpA*	−5.08
ATP synthase gamma chain	*atpG*	−3.71
ATP synthase subunit beta	*atpD*	−3.39
ATP synthase subunit b	*atpF*	−3.35
Arginine deiminase	*arcA*	−1.74
Low-molecular-weight protein-tyrosine-phosphatase	*ptpA*	−1.68
Threonine-tRNA ligase	*thrS*	−1.59
Ornithine carbamoyltransferase	*argF*	−1.47
Carbamate kinase 2	*arcC2*	−1.28
Alanine dehydrogenase 1	*ald1*	−1.27
Dihydroorotase	*pyrC*	−1.26
l-Threonine dehydratase catabolic TdcB	*tdcB*	−1.24
Alcohol dehydrogenase	*adh*	−1.23
Carbamate kinase 1	*arcC1*	−1.19
ATP synthase subunit delta	*atpH*	−1.13
DNA-binding protein HU	*hup*	−1.13
Argininosuccinate synthase	*argG*	−1.04
Formimidoylglutamase	*hutG*	−1.03
Clumping factor A	*clfA*	−1.02

a All proteins were significantly different; *P *< 0.05.

10.1128/mBio.01581-20.4 FIG S4 The expression of extracellular and intracellular is altered in S. aureus Δ*atpA* biofilm. S. aureus WT or Δ*atpA* biofilm were grown in 6-well plates for 6 days (*n* = 3 biological replicates/sample), whereupon supernatants and biofilm extracts were prepared for LC-MS/MS analysis. Significantly differentially expressed proteins (*P < *0.05) were imported into Proteomaps to generate Voronoi treemaps, where differentially expressed proteins were grouped into cellular processes and the area of the polygon reflects relative protein abundance. Download FIG S4, TIF file, 1.7 MB. Copyright © 2020 Bosch et al. 2020 Bosch et al. This content is distributed under the terms of the Creative Commons Attribution 4.0 International license.

10.1128/mBio.01581-20.9 DATA SET S1 LC-MS/MS analysis of extracellular proteins in Δ*atpA* and wild-type (WT) biofilm. Download Data Set S1, XLSX file, 0.05 MB. Copyright © 2020 Bosch et al. 2020 Bosch et al. This content is distributed under the terms of the Creative Commons Attribution 4.0 International license.

10.1128/mBio.01581-20.10 DATA SET S2 LC-MS/MS analysis of intracellular proteins in Δ*atpA* and wild-type (WT) biofilm. Download Data Set S2, XLSX file, 0.1 MB. Copyright © 2020 Bosch et al. 2020 Bosch et al. This content is distributed under the terms of the Creative Commons Attribution 4.0 International license.

### S. aureus ATP synthase contributes to biofilm persistence during orthopedic implant infection.

**FIG 6:**
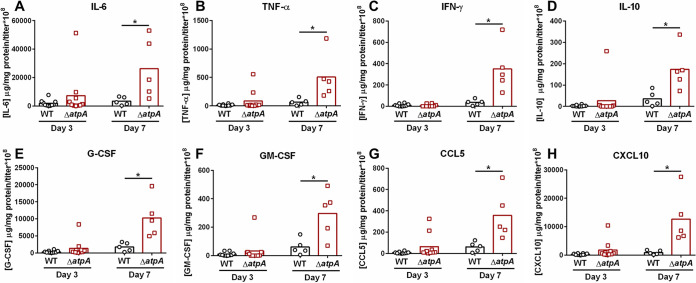
S. aureus AtpA attenuates inflammatory mediator production during prosthetic joint infection (PJI). C57BL/6NCrl mice were infected with 10^3^ CFU of S. aureus WT or Δ*atpA* using a model of PJI. Implant-associated tissue was collected at days 3 or 7 postinfection, and inflammatory mediators quantified using a multianalyte bead array. IL-6 (A), TNF-α (B), IFN-ɣ (interferon-gamma) (C), IL-10 (D), G-CSF (granulocyte colony-stimulating factor) (E), GM-CSF (granulocyte-macrophage colony-stimulating factor) (F), CCL5 (regulated upon activation T cell expressed and secreted; RANTES) (G), and CXCL10 (interferon-inducible protein 10 kDa) (H) concentrations were normalized to the protein concentration per sample and bacterial titer of each mouse to correct for differences in infectious burden between WT and Δ*atpA*. Results from day 3 represent the mean combined from 2 independent experiments (*n* = 10 mice/group) and day 7 from one experiment (*n* = 5 mice/group). Significant differences are denoted by asterisks (*, *P < *0.05; Student's *t* test with Holm-Sidak correction).

**FIG 7:**
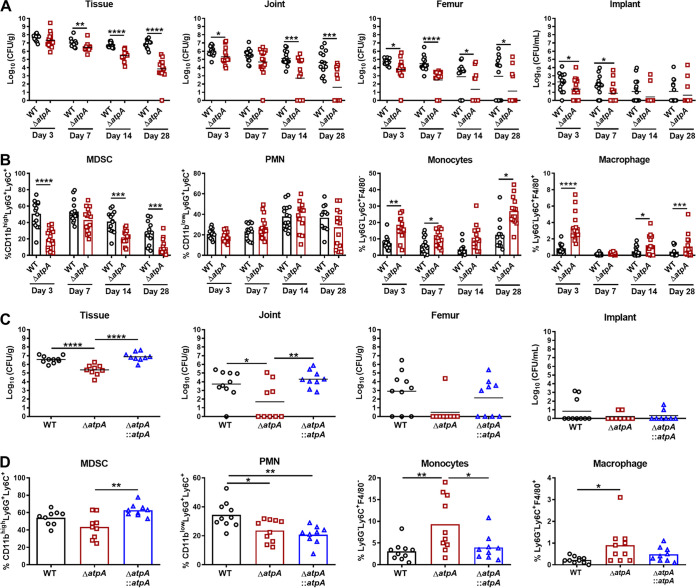
S. aureus AtpA is critical for regulating leukocyte influx and biofilm persistence. (A) C57BL/6NCrl mice were infected with 10^3^ CFU of S. aureus WT or Δ*atpA* using a model of prosthetic joint infection. Animals were sacrificed at the indicated intervals, whereupon bacterial burden in the implant-associated tissue, joint, femur, and implant was quantified with results expressed as log_10_-transformed values from 3 independent experiments (*n* = 13 to 16 mice/group). (B) Flow cytometry was performed on implant-associated tissue to quantify infiltrating leukocyte populations. (C) Bacterial burden and (D) leukocyte influx are shown for complementation studies at day 14 postinfection combined from 2 independent experiments (*n* = 9 to 10 mice/group). Significant differences are denoted by asterisks (*, *P < *0.05, **, *P < *0.01, ***, *P < *0.001, and ****, *P < *0.0001; one-way ANOVA with Tukey’s multiple-comparison test).

To elucidate the role of S. aureus ATP synthase during biofilm infection, a mouse model of PJI was utilized. To ensure an equal growth phase of WT and Δ*atpA* prior to *in vivo* inoculation, bacteria were collected in exponential phase at an optical density at 600 nm (OD_600_) of 0.25 (Fig. S5). A similar heightened inflammatory profile was observed during PJI with Δ*atpA* as was seen *in vitro*, with significantly higher levels of IL-6, TNF-α, IFN-γ, granulocyte colony-stimulating factor (G-CSF), granulocyte-macrophage colony-stimulating factor (GM-CSF), monocyte chemoattractant protein 5 (CCL5), and interferon-inducible protein 10 kDa (CXCL10) expression, primarily at day 7 postinfection (Fig. 6). Although IL-10 levels were also significantly elevated in response to Δ*atpA* (Fig. 6D), the totality of the data suggest a proinflammatory bias in response to Δ*atpA* biofilm. Additional proinflammatory mediators were also elevated in Δ*atpA*-infected mice, although these did not reach statistical significance (Fig. S6). The enhanced proinflammatory response in Δ*atpA*-infected mice coincided with reduced bacterial burden in the tissue, knee joint, and femur (Fig. 7A), concomitant with decreased MDSC and increased monocyte and MΦ infiltrates in the infected tissue (Fig. 7B) that was complementable (Fig. 7C). Furthermore, functional ATP synthase was necessary for establishing persistent PJI, as Δ*atpA*-infected mice had no detectable bacteria in implant-associated tissues at 3 months postinfection (Fig. S7).

10.1128/mBio.01581-20.5 FIG S5 Equivalent growth rates of S. aureus Δ*atpA* and WT following subculture. S. aureus WT and Δ*atpA* were grown in liquid medium at 37°C, where optical density measurements were taken for 24 h in an automated plate reader (A, left panel) or following overnight culture using a spectrophotometer (A, right panel) (*n* = 6 and 5 biological replicates, respectively). Overnight cultures were reinoculated in fresh TSB at an OD of 0.05 and subcultured for 4 h, where growth was monitored every 30 min using an automated plate reader (B, left panel) or at 4 h in a spectrophotometer (B, right panel) (*n* = 1 and 3 biological replicates, respectively). Significant differences are denoted by asterisks (****, *P < *0.0001; Student’s *t*-test). Download FIG S5, TIF file, 0.2 MB. Copyright © 2020 Bosch et al. 2020 Bosch et al. This content is distributed under the terms of the Creative Commons Attribution 4.0 International license.

10.1128/mBio.01581-20.6 FIG S6 Effect of S. aureus AtpA on inflammatory mediator production during prosthetic joint infection (PJI). C57BL/6NCrl mice were infected with 10^3^ CFU of S. aureus WT or Δ*atpA* using a model of PJI. Implant-associated tissue was collected at days 3 or 7 postinfection and inflammatory mediators quantified using a multianalyte bead array. IL-12p70 (A), IL-12p40 (B), IL-1α (C), IL-1β (D), IL-17 (E), CCL2 (monocyte chemoattractant protein 2) (F), CXCL1 (growth-related oncogene alpha) (G), and CXCL2 (macrophage inflammatory protein 2) (H) concentrations were normalized to the protein concentration per sample and bacterial titer of each mouse to correct for differences in infectious burden between WT and Δ*atpA*. Results from day 3 represent the mean combined from 2 independent experiments (*n* = 10 mice/group) and day 7 from one experiment (*n* = 5 mice/group). No significant differences between groups were detected by a Student’s *t*-test (α = 0.05). Download FIG S6, TIF file, 0.2 MB. Copyright © 2020 Bosch et al. 2020 Bosch et al. This content is distributed under the terms of the Creative Commons Attribution 4.0 International license.

10.1128/mBio.01581-20.7 FIG S7 S. aureus ATP is critical for establishing persistent prosthetic joint infection (PJI). C57BL/6NCrl mice (*n*= 10/group) were infected with 10^3^ CFU of S. aureus WT or Δ*atpA* using a model of PJI. Mice were sacrificed at 3 months postinfection, whereupon bacterial burden in the implant-associated tissue, joint, and femur was quantified. Results represent the mean combined from 2 independent experiments (*n* = 10 mice/group). Significant differences are denoted by asterisks (****, *P < *0.0001; Student’s *t*-test). Download FIG S7, TIF file, 0.1 MB. Copyright © 2020 Bosch et al. 2020 Bosch et al. This content is distributed under the terms of the Creative Commons Attribution 4.0 International license.

### Inhibiting cell lysis in Δ*atpA* attenuates leukocyte proinflammatory responses.

**FIG 8:**
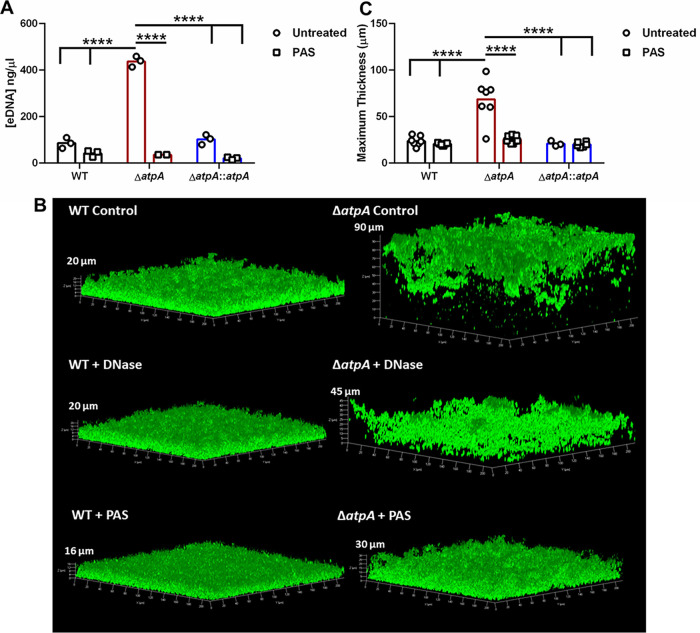
Inhibiting bacterial lysis negates the aberrant morphology and eDNA levels of S. aureus Δ*atpA* biofilm. (A) Biofilms were grown in 6-well plates in the presence of PAS (10 μg/ml), and eDNA concentrations were quantified at day 3 (*n* = 2 to 3 biological replicates). (B) GFP-expressing WT or Δ*atpA* were grown in 8-well chamber slides and treated with DNase (100 U/ml) or PAS (10 μg/ml) at the time of biofilm inoculation and throughout the 4-day maturation period, whereupon images were acquired by confocal laser scanning microscopy. (C) Maximum thickness of biofilms was calculated using Comstat 2 combined from 1 to 3 independent experiments (*n* = 3 to 9 biological replicates). Significant differences are denoted by asterisks (****, *P < *0.0001; two-way ANOVA with Tukey’s multiple-comparison test).

**FIG 9:**
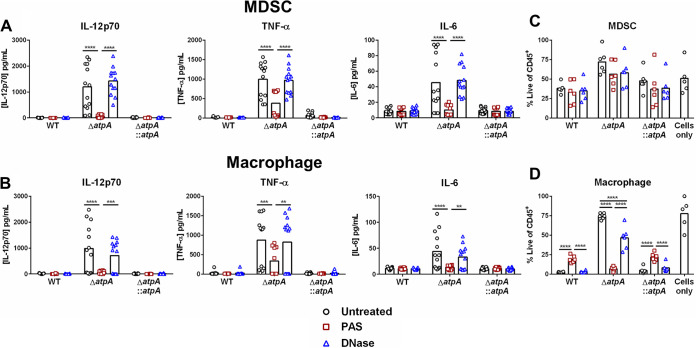
AtpA-dependent inhibition of leukocyte cytokine production is cell lysis-dependent. Bone marrow-derived MDSCs (A) or macrophages (B) were cocultured for 2 h with biofilm treated with DNase (100 U/ml) or PAS (10 μg/ml), whereupon supernatants were analyzed using a mouse cytometric bead array inflammation kit (results represent the mean combined from 3 independent experiments; *n* = 13 to 16 biological replicates). Viability of MDSCs (C) and macrophages (D) following PAS or DNase treatment with results presented as the percentage of live CD45^+^ leukocytes (*n* = 5 to 6 biological replicates from 2 independent experiments). Significant differences are denoted by asterisks (**, *P* < 0.01, ***, *P* < 0.001, and ****, *P* < 0.0001; two-way ANOVA with Tukey’s multiple-comparison test).

We next investigated potential mechanisms for the unique structure of Δ*atpA* biofilm that may account for its ability to enhance leukocyte cytokine release. A major component of the extracellular polymeric substance (EPS) of biofilm is eDNA, which is released by the lysis of a subset of bacterial cells within the biofilm (32). Since eDNA levels were significantly increased in Δ*atpA* biofilm (Fig. 3B), we examined whether inhibiting biofilm lysis with polyanethole sodium sulfanate (PAS), which blocks the major S. aureus autolysin Atl (33), would reverse the heightened inflammatory response elicited by Δ*atpA*. PAS treatment of Δ*atpA* biofilm significantly reduced eDNA levels (Fig. 8A), resulting in a more compact structure (Fig. 8B and C). PAS had little effect on WT biofilm, both in terms of morphology and eDNA release (Fig. 8). Treatment with DNase partially restored Δ*atpA* biofilm structure (Fig. 8B), suggesting the involvement of other cell lysis-dependent factors. Overall, these data suggest that enhanced eDNA release partially contributes to the altered structure of Δ*atpA* biofilm, which can be reversed by inhibiting cell lysis. PAS treatment also diminished the enhanced proinflammatory response of MDSCs and MΦs to Δ*atpA* biofilm (Fig. 9). Even though DNase treatment partially restored biofilm structure, it did not attenuate cytokine production elicited by Δ*atpA* biofilm (Fig. 9), revealing that leukocyte activation is driven by cell lysis-dependent factors other than eDNA in agreement with the findings with MyD88^−/−^ leukocytes (Fig. 4). PAS treatment significantly reduced macrophage viability following coculture with Δ*atpA* biofilm (Fig. 9D), in agreement with its ability to diminish cytokine production to levels observed with WT biofilm (Fig. 9B). A similar trend was observed with MDSCs, although this did not reach statistical significance (Fig. 9C). Macrophage viability was also reduced during coculture with DNase-treated Δ*atpA* biofilm, but this was less dramatic than PAS (Fig. 9D).

### Deletion of the major S. aureus autolysin Atl reverses heightened proinflammatory cytokine release from leukocytes in response to *ΔatpA* biofilm.

**FIG 10:**
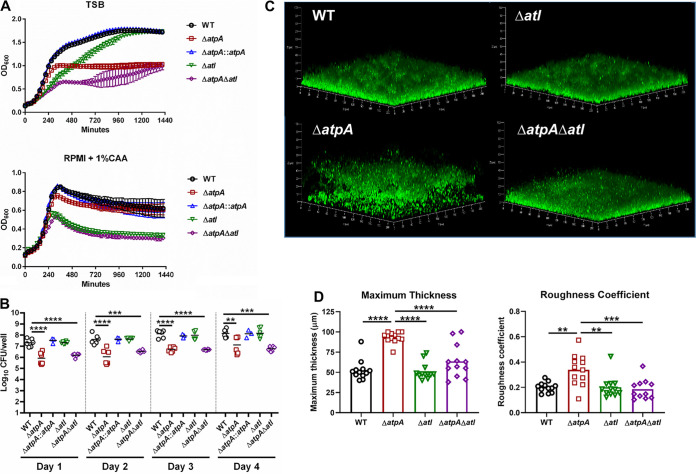
Atl deletion reverses the aberrant morphology of S. aureus Δ*atpA* biofilm. (A) OD_600_ measurements of S. aureus strains in tryptic soy broth (TSB) or RPMI-1640 supplemented with 1% Casamino Acids (CAA) (*n* = 6 biological replicates). (B) CFU of *in vitro* biofilms at various stages of maturation (*n* = 3 to 6 biological replicates). (C) Representative 3D images of 4-day-old biofilm acquired using confocal laser scanning microscopy. (D) Maximum thickness and roughness coefficient measurements were calculated by Comstat 2 analysis combined from 2 independent experiments (*n* = 12 biological replicates). Significant differences are denoted by asterisks (**, *P < *0.01, ***, *P < *0.001, and ****, *P < *0.0001; one-way ANOVA with Dunnett’s multiple-comparison test with WT control [B] or one-way ANOVA with Tukey’s multiple-comparison test [D]).

**FIG 11:**
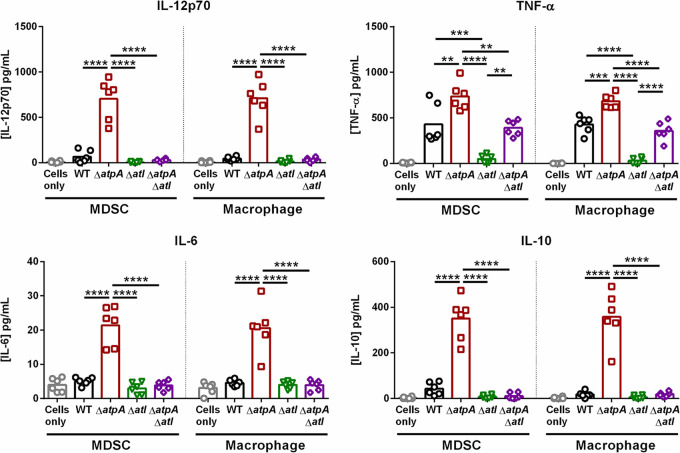
Atl deletion prevents the enhanced cytokine response elicited by S. aureus Δ*atpA* biofilm. Bone marrow-derived MDSCs or macrophages were cocultured with biofilm for 2 h, whereupon supernatants were analyzed using a mouse cytometric bead array inflammation kit. Results represent the mean combined from 2 independent experiments (*n* = 6 biological replicates). Significant differences are denoted by asterisks (**, *P < *0.01, ***, *P < *0.001, and ****, *P < *0.0001; one-way ANOVA with Tukey’s multiple-comparison test).

A main target of PAS in staphylococci is autolysins (33), and the major S. aureus autolysin, Atl, plays a role in cell wall turnover, division, and biofilm formation (34, 35). Since PAS treatment attenuated leukocyte proinflammatory cytokine production in response to Δ*atpA* biofilm, we constructed a double-mutant strain (Δ*atpA* Δ*atl*) to assess the role of Atl-mediated cell lysis in Δ*atpA* biofilm. As expected, both *atpA* and *atl* were critical for S. aureus growth in broth and biofilm culture, and the Δ*atpA*Δ*atl* strain exhibited attenuated growth under both conditions (Fig. 10A and B). However, the diffuse biofilm structure of Δ*atpA* was reversed in Δ*atpA*Δ*atl* with a significant reduction in the maximum thickness and roughness coefficients (Fig. 10C and D). Atl-mediated lysis also contributed to the enhanced cytokine production by MDSCs and MΦs in response to Δ*atpA* biofilm since this was significantly reduced in Δ*atpA*Δ*atl* (Fig. 11). Taken together, our findings demonstrate that the increased inflammatory properties of MDSCs and MΦs cocultured with Δ*atpA* biofilm is a lysis-dependent phenotype since the chemical inhibition of cell lysis or Atl deletion dampens leukocyte cytokine production.

## DISCUSSION

In the present study, a screen of the NTML identified a role for S. aureus ATP synthase in attenuating MDSC and MΦ cytokine production, and S. aureus Δ*atpA* was cleared in a mouse model of PJI, demonstrating the importance of ATP synthase in biofilm persistence. This report provides a link between bacterial ATP synthase activity and host immunity during biofilm development, which is largely influenced by increased bacterial cell lysis.

Previous studies have shown that the inactivation of S. aureus ATP synthase leads to increased susceptibility to polymyxins, gentamicin, and nitric oxide (26, 36, 37). ATP synthase is the primary energy generator for cellular respiration, so it was not unexpected that *atpA* disruption affected S. aureus growth in both planktonic and biofilm conditions. Interestingly, Δ*atpA* biofilm elicited heightened proinflammatory cytokine production in mouse MDSCs and MΦs, and similar results were obtained with human monocyte-derived MΦs, demonstrating the translational relevance of these findings. The increase in cytokine production likely resulted from improved leukocyte viability based on the reductions in toxin and protease production by Δ*atpA* biofilm. Of note, a prior screen of the NTML identified *atpA* as important for attenuating macrophage proinflammatory cytokine production in response to planktonic S. aureus (38), revealing the broader implications for bacterial ATP synthase-dependent mechanisms in dictating leukocyte activation.

The essential role of S. aureus ATP synthase in influencing the host inflammatory response during biofilm formation *in vivo* was demonstrated by the finding that Δ*atpA* was cleared in a mouse PJI model at 3 months postinfection. However, it is important to emphasize the differential involvement of S. aureus ATP synthase during biofilm versus nonbiofilm infections. For example, a recent study by Grosser et al. in a mouse SSTI model demonstrated that S. aureus Δ*atpG* was cleared within 3 days (26), whereas in the current study, Δ*atpA* was still detected at day 28 postinfection in a mouse PJI biofilm model. This highlights the distinctions in S. aureus persistence between biofilm versus acute tissue infection, and the metabolic state of bacteria in each setting may explain the differential survival of S. aureus ATP synthase mutants.

S. aureus biofilms exhibit metabolic heterogeneity, with a subpopulation of metabolically dormant organisms (39, 40). Therefore, it is conceivable that this population of cells is more recalcitrant to the loss of S. aureus ATP synthase, enabling their increased survival in the host. This is supported by a recent study demonstrating that ATP depletion is responsible for promoting antibiotic-tolerant persister cells in S. aureus (41). In contrast, S. aureus is metabolically active during acute tissue infection, which may explain why bacteria are more sensitive to the loss of respiratory capacity and rapidly cleared. Indeed, the reduced fitness of Δ*atpG* was attributed, in part, to a failure in intracellular acidification, which is required for the optimal activity of fermentative enzymes that generate energy in the face of respiration defects (26). A critical role for S. aureus *atpA* in attenuating the host immune response *in vivo* was demonstrated by the finding that Δ*atpA* elicited heightened proinflammatory mediator production in a mouse model of S. aureus PJI. This coincided with a significant reduction in MDSCs concomitant with increased monocyte and MΦ recruitment, a relationship that our prior studies have established coincides with biofilm clearance (18, 20, 22, 27, 42).

We hypothesized that the diffuse structure of Δ*atpA* biofilm might enable better recognition of S. aureus PAMPs leading to increased cytokine production, which was suggested by elevated eDNA levels in Δ*atpA* biofilm. However, this seems unlikely, since cytokine levels were equivalent in MyD88^−/−^ and WT leukocytes following coculture with Δ*atpA* biofilm, although a role for MyD88-independent pathways cannot be disregarded (i.e., nucleotide-binding and oligomerization domain [NOD] receptors). In terms of TLR9 involvement, this was not unexpected since TLR9 is an endosomal receptor (43) and MΦs fail to phagocytose S. aureus biofilm (42), which would prevent eDNA from triggering TLR9 intracellularly. Furthermore, treatment of Δ*atpA* biofilm with DNase did not attenuate enhanced cytokine release by MDSCs or MΦs.

A prior study from our laboratory revealed a critical role for bacterial lysis in blocking MΦ phagocytosis in response to S. aureus biofilm (42). In the current report, we found that inhibiting lysis of S. aureus Δ*atpA* using two independent approaches, namely, PAS treatment or a Δ*atpAΔatl* strain, negated the enhanced proinflammatory cytokine response by MDSCs and MΦs. This finding suggests that factors released following Δ*atpA* biofilm lysis are responsible for promoting leukocyte proinflammatory activity. Additionally, LC-MS/MS analysis revealed a significant reduction in numerous toxins and proteases in Δ*atpA* compared to WT biofilm. Among the proteins that were significantly reduced in Δ*atpA* biofilm were serine proteases of the Spl family (SplB, SplC, SplE, and SplF) and aureolysin, in addition to several toxins, such as Hla and leukocidin-like proteins. It is well established that Hla and leukocidins induce leukocyte lysis by binding to specific immune receptors (2, 44–46), which suggested that the reduction in these virulence factors in Δ*atpA* biofilm may be responsible for the increased viability of MDSCs and MΦs. Indeed, this was observed and suggested that the increase in proinflammatory cytokines in response to Δ*atpA* biofilm resulted from a larger number of viable leukocytes that continued to produce cytokines. This highlights the critical role of toxins targeting leukocyte survival, in agreement with earlier reports (47). Our recent study identified many of the same proteins to be responsible for inhibiting MΦ phagocytosis in response to S. aureus biofilm (42).

It remains unclear what combination of factors is responsible for inhibiting leukocyte proinflammatory responses to WT biofilm as demonstrated in this study. The metabolic deficit following the loss of ATP synthase in S. aureus likely impinges on multiple pathways, making the ability to pinpoint the phenotypes to one factor unlikely. Nevertheless, this study highlights a previously unappreciated role for ATP synthase in modulating the host immune response to S. aureus biofilm and infection persistence.

## MATERIALS AND METHODS

### Animals.

C57BL/6NCrl (RRID IMSR_CRL:27), MyD88^−/−^ (RRID IMSR_JAX:009088), and TLR2^−/−^ mice (RRID IMSR_JAX:022507) were bred in-house at the University of Nebraska Medical Center (UNMC), and mice of the same sex were randomized into standard-density cages upon weaning (*n* = 5 animals per cage). Mice were housed in a restricted-access biosafety level 2 (BSL2) room equipped with ventilated microisolator cages and maintained at 21°C under a 12-h light:12-h dark cycle with *ad libitum* access to water and chow with nestlets provided for enrichment. This study was conducted in strict accordance with the recommendations in the Guide for the Care and Use of Laboratory Animals of the National Institutes of Health. The protocol was approved by the UNMC Institutional Animal Care and Use Committee (18-013-03).

### S. aureus strains.

The strains used in this study are described in Table S1. The S. aureus clinical isolate USA300 LAC 13C was cured of the LAC-p03 erythromycin (erm) resistance plasmid (23, 48) and is referred to as WT. The USA300 JE2 NTML strain *ΔatpA* was moved to the LAC USA300 13C background via ϕ11 transduction as previously described (23). Strain background was validated by plasmid purification, and Δ*atpA* was confirmed by growth on erm plates and PCR using atpA_fwd and atpA_rev primers (Table S1).

10.1128/mBio.01581-20.8 TABLE S1 Bacterial strains and primers used in this study. Download Table S1, DOCX file, 0.02 MB. Copyright © 2020 Bosch et al. 2020 Bosch et al. This content is distributed under the terms of the Creative Commons Attribution 4.0 International license.

Chromosomal complementation of Δ*atpA* was performed by replacing the transposon for the native gene using the allelic exchange plasmid pJB38 as previously described (49). Briefly, the *atpA* gene flanked with 1-kb arms was amplified using ATPase_alpha_C_fwd and ATPase_alpha_C_rev primers, and the shuttle vector was amplified using pJB38_fwd and pJB38_rev primers (Table S1). The resulting fragments were assembled using the NEBuilder HiFi DNA assembly cloning kit (New England Biolabs) to generate the pAQ67 plasmid that was electroporated into E. coli E10B. Subsequently, pAQ67 was electroporated into S. aureus RN4220 followed by transduction into Δ*atpA* using ϕ11 to perform the allelic exchange process (49, 50). The Δ*atpA*Δ*atl* strain was constructed by ϕ11 transduction of the NTML *atpA* mutation into a USA300 LAC 13C *atl* clean deletion mutant (Δ*atl*) (51), with *atpA* and *atl* loss verified by PCR. To visualize biofilm development, bacterial strains were transduced with pCM29-green fluorescent protein (GFP) (52) using ϕ11 and confirmed by chloramphenicol resistance.

### S. aureus planktonic and biofilm growth.

S. aureus strains were grown on Trypticase soy agar (TSA) with 5% sheep blood 1 day prior to the inoculation of broth cultures. For *in vitro* biofilm experiments, single colonies were added to 5 ml of RPMI-1640 supplemented with 1% CAA, 1% l-glutamine, and 1% HEPES (referred to as biofilm medium) and grown overnight at 37°C with constant shaking at 250 rpm for 16 to 18 h prior to use. Overnight cultures were diluted to an OD_600_ of 0.05 for inoculation into 96-well and 12-well plates, or 8-well glass-bottom chamber slides (Thermo Fisher Nunc) that were previously coated with 20% human plasma in carbonate-bicarbonate buffer overnight at 4°C. Chloramphenicol (5 μg/ml) was added to biofilm medium for maintenance of the pCM29-GFP plasmid. Static biofilms were grown at 37°C with approximately 50% of medium replaced every 24 h. Where indicated, biofilms were treated with 100 U DNase or 10 μg/ml of PAS beginning at the time of biofilm inoculation to assess the role of extracellular DNA or cell lysis, respectively, on MDSC and MΦ inflammatory properties.

Growth rates of S. aureus strains in liquid medium were determined using an Infinite Pro 200 (Tecan). Static biofilms were visualized using a Zeiss 710 META laser scanning confocal microscope (Carl Zeiss) at ×40 magnification. To obtain a representation of biofilm development and structure, z-stack images (0.88-μm sections) were collected from 2 to 3 biological replicates (wells) for each strain, with results confirmed in 2 to 3 independent experiments. Maximum thickness and the dimensionless roughness coefficient of biofilms was determined using Comstat 2 (ImageJ) (53–55).

### MDSC and MΦ cultures.

Primary bone marrow-derived MDSCs and MΦs were prepared from C57BL/6, MyD88^−/−^, or TLR2^−/−^ mice as previously described (19, 22, 56). MDSCs were expanded for 4 days in RPMI-1640 supplemented with 10% fetal bovine serum (FBS), 1% l-glutamine, 1% HEPES, 1% antibiotic-antimitotic, 50 μM beta-mercaptoethanol, 40 ng/ml GM-CSF, and 40 ng/ml G-CSF with 40 ng/ml IL-6 added at day 3 of culture. Following expansion, MDSCs were purified using an anti-Ly6G microbead kit (Miltenyi Biotec). MΦs were propagated for 7 days in RPMI-1640 supplemented with 10% FBS, 1% l-glutamine, 1% HEPES, 1% antibiotic-antimitotic, 50 μM beta-mercaptoethanol, and 10% conditioned medium from L929 fibroblasts as a source of macrophage colony-stimulating factor (M-CSF) (28, 57). For visualizing MΦ invasion into biofilm by confocal microscopy, MΦs were stained with CellTracker deep red (1 μM; Invitrogen) according to the manufacturer’s instructions.

Human monocytes were obtained from healthy human donors by the UNMC Elutriation Core Facility by countercurrent centrifugal elutriation, in full compliance and with approval of the Institutional Review Board (IRB). Cells were cultured at 1 × 10^6^ cells/ml in RPMI-1640 supplemented with recombinant human M-CSF, 10% human serum, and 1% antibiotic-antimitotic for 7 days until harvest for experiments.

### Quantification of cytokine production by leukocytes following biofilm coculture.

MDSCs and MΦs (5 × 10^4^/well) were cocultured with biofilm for 2 h at 37°C in a 96-well plate, whereupon plates were centrifuged and supernatants stored at −20°C until analysis. Cytokine production was quantified using BD cytometric bead array mouse (catalog no. 552364) and human (catalog no. 551811) inflammation kits (both from BD Biosciences) according to the manufacturer’s instructions and analyzed by flow cytometry using a BD LSR II.

### Gentamicin protection assay.

To determine whether S. aureus Δ*atpA* was more susceptible to MΦ killing, a gentamicin protection assay was utilized. Overnight cultures of WT, Δ*atpA*, and Δ*atpA::atpA* were washed 1 time with PBS and incubated with MΦs at a multiplicity of infection (MOI) of 1:1, 5:1, and 10:1 (bacteria:MΦ) in a 96-well plate for 1 h at 37°C to allow for phagocytosis. After 1 h, plates were centrifuged, and fresh medium containing 100 μg/ml gentamicin was added for 30 min at 37°C to kill residual extracellular bacteria. Next, fresh medium containing low-dose gentamicin (1 μg/ml) was added, and MΦs were incubated for various intervals over a 24-h period. At the indicated time points, MΦs were lysed with 100 μl sterile H_2_O followed by serial dilution on blood agar plates to quantify intracellular bacterial burden.

### Orthopedic implant model.

To evaluate the importance of *atpA* during biofilm development *in vivo*, a mouse model of S. aureus PJI was used as previously described (58). Since Δ*atpA* had a postexponential-phase growth defect in TSB compared to WT, cultures were grown overnight at 37°C at 250 rpm and reinoculated at a starting OD_600_ of 0.05 the following day and allowed to replicate for 4 h. There was no significant difference in the growth rate or number of viable bacteria following the 4-h subculture (Fig. S5). Sex- and age-matched C57BL/6NCrl mice (8 to 10 weeks old) were anesthetized with a ketamine/xylazine cocktail, and a medial parapatellar arthrotomy was performed to expose the distal femur. A burr hole was created in the femoral intercondylar notch using a 26-gauge needle, whereupon a 0.8-cm-long orthopedic-grade Kirschner wire (0.6 mm diameter, Nitinol [nickel-titanium]; Custom Wire Technologies) was inserted into the intramedullary canal, leaving approximately 1 mm of the wire protruding into the joint space. Approximately 10^3^ CFU of either WT, Δ*atpA*, or Δ*atpA::atpA* was inoculated into the joint cavity, with inocula verified the following day after growth on blood agar. The surgical site was sutured closed, and Buprenex (Reckitt Benckiser Health Care) was administered immediately following surgery and 24 h later for pain relief. Animals did not display any ambulatory defects or pain behaviors after this period and exhibited normal activity.

### Flow cytometry.

Leukocyte infiltrates into the surrounding soft tissue following S. aureus PJI were characterized using flow cytometry as previously described (59). Briefly, the soft tissue surrounding the knee joint was excised, disrupted using the blunt end of a 3-ml syringe plunger in PBS containing protease inhibitor (Thermo Scientific, Rockford, IL), and passed through a 70-μm filter. Red blood cells (RBCs) were lysed using RBC lysis buffer (BioLegend, San Diego, CA), and the single-cell suspension was stained with CD11b-fluorescein isothiocyanate (FITC), CD45-allophycocyanin (APC), Ly6G-phycoerythrin (PE), Ly6C-peridinin chlorophyll protein (PerCP)-Cy5.5, F4/80-PE-Cy7 (BioLegend and BD Biosciences, San Diego, CA), and a Live/Dead fixable blue dead cell stain kit (Invitrogen, Eugene, OR) according to the manufacturers’ instructions. Cell populations were analyzed using a BD LSR II and FACSDiva software (BD Bioscience, San Jose, CA), where MDSCs (CD11b^high^ Ly6G^+^ Ly6C^+^ F4/80^−^), neutrophils (CD11b^low^ Ly6G^+^ Ly6C^+^ F4/80^−^), monocytes (Ly6G^−^ Ly6C^+^ F4/80^−^), and MΦs (Ly6G^−^ Ly6C^−^ F4/80^+^) are reported as the percentage of live CD45^+^ cells.

### Multianalyte microbead array.

To quantify inflammatory mediator expression associated with WT and Δ*atpA* PJI, homogenates prepared from the soft tissue surrounding the infected joint were analyzed using a Milliplex MAP mouse cytokine/chemokine magnetic bead panel (catalog no. MCYTMAG-70K-PX32; Millipore Sigma, Billerica, MA). Results were normalized to the total protein concentration per sample and bacterial burden to adjust for the differences in titer between WT and Δ*atpA*-infected animals.

### Mass spectrometry.

The conditioned medium and cell extracts from WT and Δ*atpA* biofilm were evaluated by LC-MS/MS to compare changes in the extracellular and intracellular proteome, respectively. WT and Δ*atpA* biofilm were grown in 6-well plates as described above, whereupon supernatants were collected, centrifuged at 14,000 rpm for 10 min, and passed through a 0.2-μm filter to remove any bacterial cells, followed by vacuum centrifugation to concentrate extracellular proteins. Biofilms were disrupted in cell lysis buffer (1× PBS supplemented with 1× protease inhibitor and one-half phosphatase inhibitor tablet [both from Thermo Fisher Scientific]) and lysed using a bead beater (Bullet Blender, Next Advance), and cell membranes were removed by centrifugation at 14,000 rpm for 10 min.

The protein concentration for each sample was determined using a bicinchoninic acid (BCA) protein assay kit (Pierce). Protein digestion for mass spectrometry and tandem mass tag (TMT) labeling of peptides were conducted following the manufacturer’s recommendations. Briefly, 100 μg of protein from each sample was reconstituted to 100 μl with 100 mM triethylammonium bicarbonate (TEAB). Proteins were next reduced with 5 μl of 200 mM tris (2-carboxyethyl) phosphine (TCEP) (1 h incubation at 55°C) and alkylated with 5 μl of 375 mM iodoacetamide (IAA) for 30 min in the dark at room temperature (RT). The reduced and alkylated proteins were purified with acetone precipitation at −20°C overnight. The protein precipitates were collected by centrifugation at 8,000 × *g* for 10 min at 4°C, and the pellets were air-dried and resuspended in 100 μl of 50 mM TEAB. Next, proteins were digested for 24 h at 37°C using 2.5 μg of trypsin per sample. The amount of peptide yield in each sample was determined using a Pierce colorimetric peptide assay kit. The amounts of peptides to be tagged were normalized and mixed with 41 μl of TMT reagent (TMTsixplex; Thermo Fisher Scientific) freshly dissolved in acetonitrile (ACN, 20 μg/μl) for 1 h at RT, and the reaction was quenched with 8 μl of 5% hydroxylamine (15 min incubation at RT). Tagged tryptic peptides were pooled and concentrated to ∼20 μl by vacuum centrifugation and analyzed using a high-resolution mass spectrometry nano-LC-MS/MS Tribrid system (Orbitrap Fusion Lumos coupled with an UltiMate 3000 high-performance liquid chromatography (HPLC) system; Thermo Scientific).

Approximately 800 ng of peptides were run on pre- (Acclaim PepMap 100, 75 μm by 2 cm; nanoViper) and analytical columns (Acclaim PepMap RSLC, 75 μm by 50 cm, nanoViper; both from Thermo Scientific). Peptides were eluted using a 125-min linear gradient of ACN (4 to 45%) in 0.1% fluorescent antibody (FA) and introduced to the mass spectrometer with a nanospray source. The MS scan was performed using the following detector settings: Orbitrap resolution, 120,000; scan range, 375 to 1,500 *m/z*; replicative-form (RF) lens, 60%; automatic gain control (AGC) target, 5.0E^5^; and maximum injection time, 150 ms. Ions with an intensity higher than 5.0E^3^ and a charge state of 2 to 7 were selected in the MS scan for further fragmentation. MS2 scan parameters included collision-induced dissociation (CID) collision energy, 35%; activation Q, 0.25; AGC target, 1.0E^4^; and maximum injection time, 150 ms. MS3 scan parameters were high-energy collisional dissociation (HCD) collision energy, 65%; Orbitrap resolution, 50,000; scan range, 100 to 500 *m/z*; AGC target, 1.0E^5^, and maximum injection time, 200 ms.

All MS- and sequential mass spectrometry (MS^n^)-collected spectra were analyzed using a Protein Discoverer pipeline (version 2.1; Thermo Fisher Scientific). SEQUEST HT was used to search the Swiss-Prot database (selected for S. aureus, 2019_03; 11,082 entries) using the following parameters: enzyme, trypsin; maximum missed cleavage, 2; precursor mass tolerance, 10 ppm; peptide tolerance, ±0.02 Da; fixed modifications (carbamidomethyl [C] and TMTsixplex [any N terminus]); and dynamic modifications (oxidation [M] and TMTsixplex [K]). The parameters for reporter ions quantifier were assigned as follows: integration tolerance, 20 ppm; integration method, most confident centroid; mass analyzer, FTMS (Fourier transform mass spectrometry); MS order, MS3; activation type, HCD; minimum collision energy, 0; and maximum collision energy, 1,000. A percolator was used to calculate the false discovery rate (FDR) for the peptide spectral matches using the following parameters: target FDR (strict), 0.01; target FDR (relaxed), 0.05; and validation based on *q* value. Quantification parameters were set as follows: peptides to use, unique; and normalization mode, total peptide amount. The complete set of differentially expressed proteins is presented in Data Set S1 and S2 in the supplemental material. Proteomaps (60) was used to generate Voronoi treemaps to visualize differentially expressed proteins in biofilm supernatant and extracts (Fig. S4).

### Extracellular DNA quantification.

eDNA isolation from static biofilms grown in 6-well plates was performed as described previously (61). Briefly, after 6 days of growth, biofilms were chilled to 4°C, 50 mM EDTA was added to the supernatant, and biofilms were mechanically disrupted in TES Buffer (Tris-HCl, pH 8.0, with 500 mM NaCl). Samples were subjected to subsequent phenol:chloroform:isoamyl alcohol (25:24:1) and chloroform:isoamyl alcohol (24:1) extractions and stored overnight at −20°C in 10% 3 M sodium acetate in EtOH. The next day, eDNA was pelleted by centrifugation and washed prior to resuspension in Tris-EDTA (TE) buffer. For eDNA quantification, qPCR for *gyrA* was performed using LightCycler DNA Master SYBR Green I (Roche).

### Statistics.

Significant differences were determined using a one- or two-way analysis of variance (ANOVA) with Tukey’s or Dunnett’s multiple comparisons, apart from the *in vivo* studies and gentamicin protection assay where significance between two groups was determined by a Student's *t* test with Holm-Sidak correction using GraphPad Prism version 6.04. Outliers were identified using a ROUT test (Q = 1%) in Prism. For all analyses, *P < *0.05 was considered statistically significant.
