# Supplementary figures and images for: Staphylococcus aureus ATP Synthase Promotes Biofilm Persistence by Influencing Innate Immunity
Source: mBio. 2020 Sep 8;11(5):e01581-20. doi: 10.1128/mBio.01581-20 (PMC7482063; doi:10.1128/mBio.01581-20)

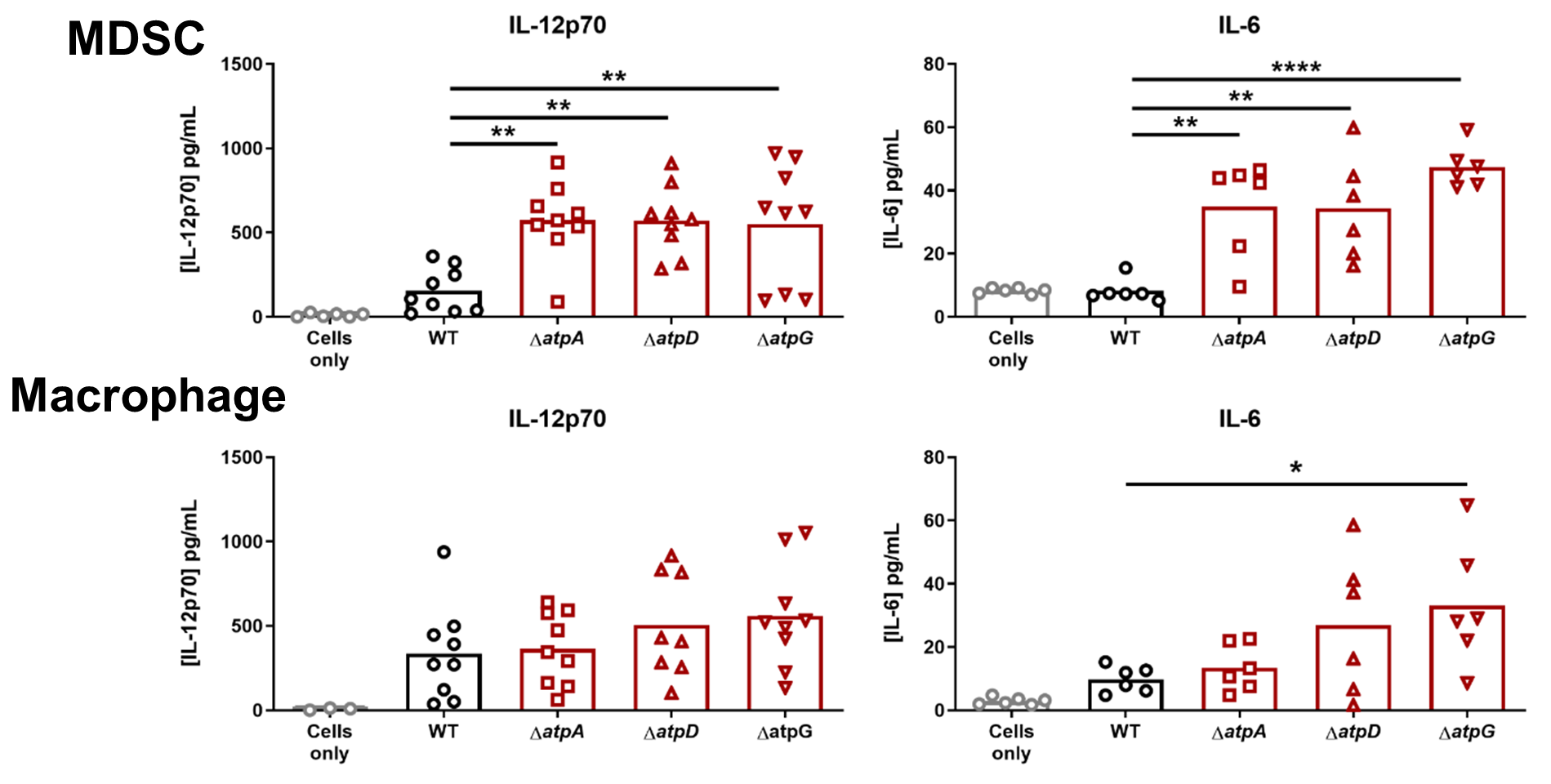

Supplement: FIG S1 [file mBio.01581-20-sf001.tif]

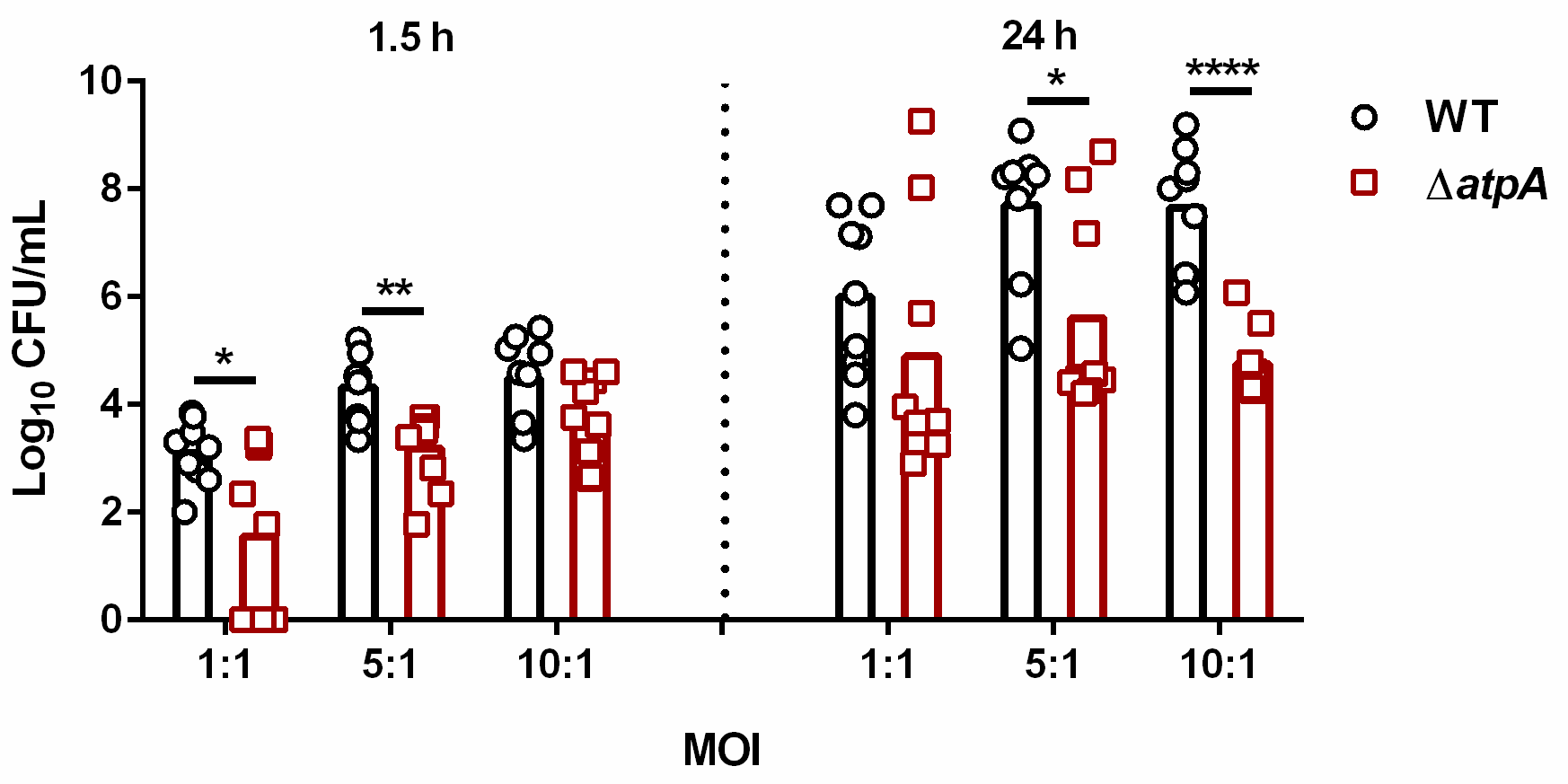

Supplement: FIG S2 [file mBio.01581-20-sf002.tif]

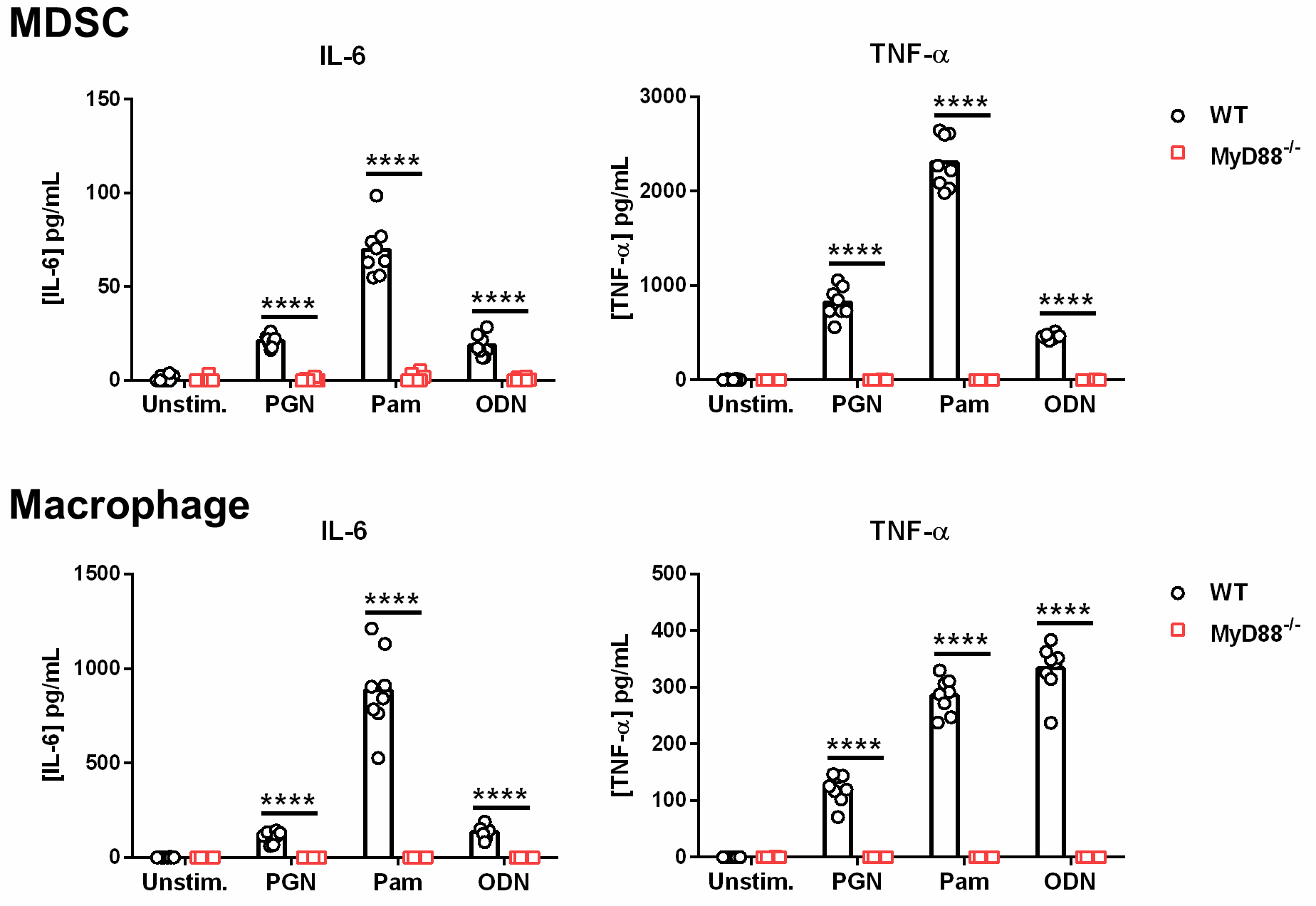

Supplement: FIG S3 [file mBio.01581-20-sf003.tif]

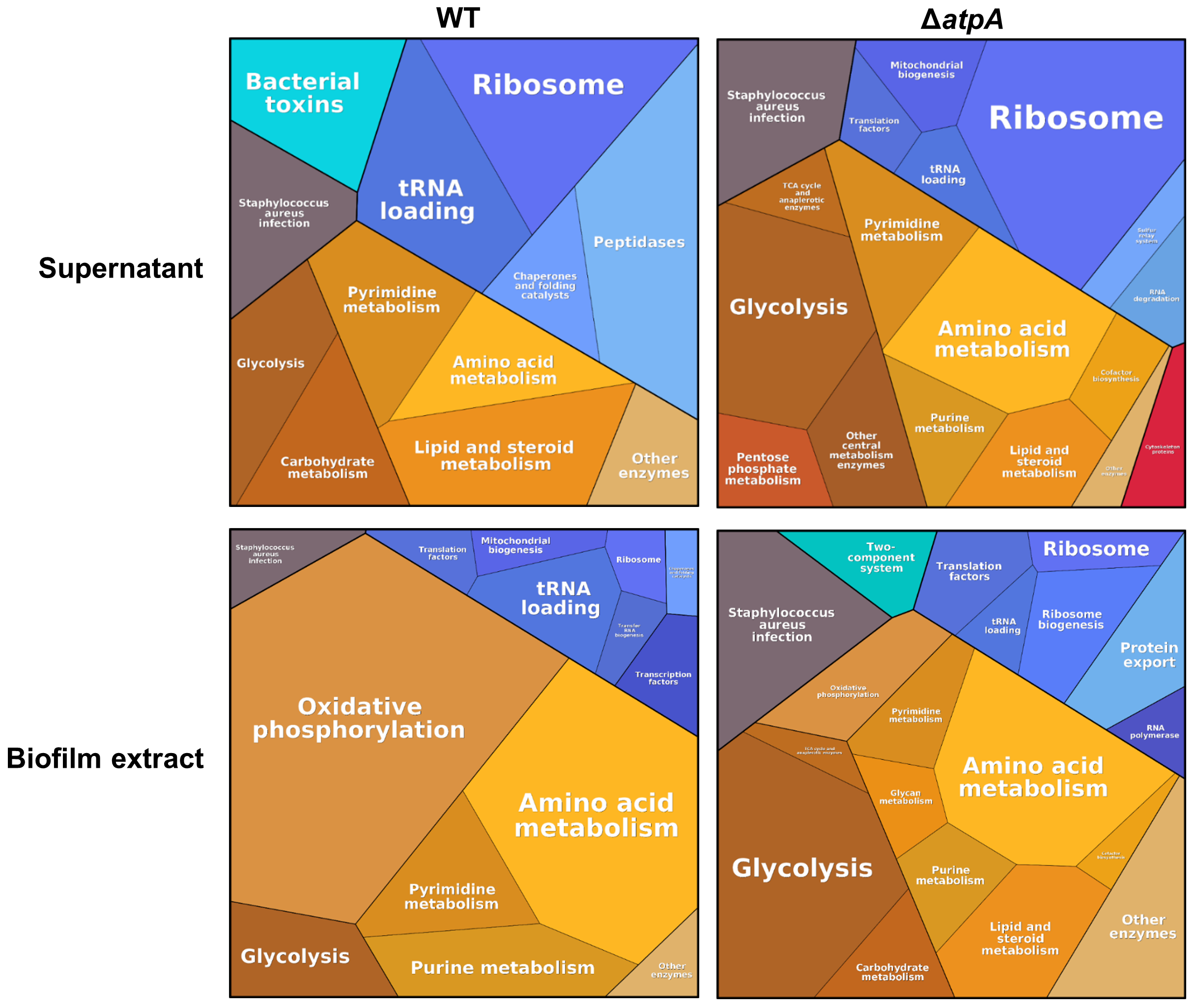

Supplement: FIG S4 [file mBio.01581-20-sf004.tif]

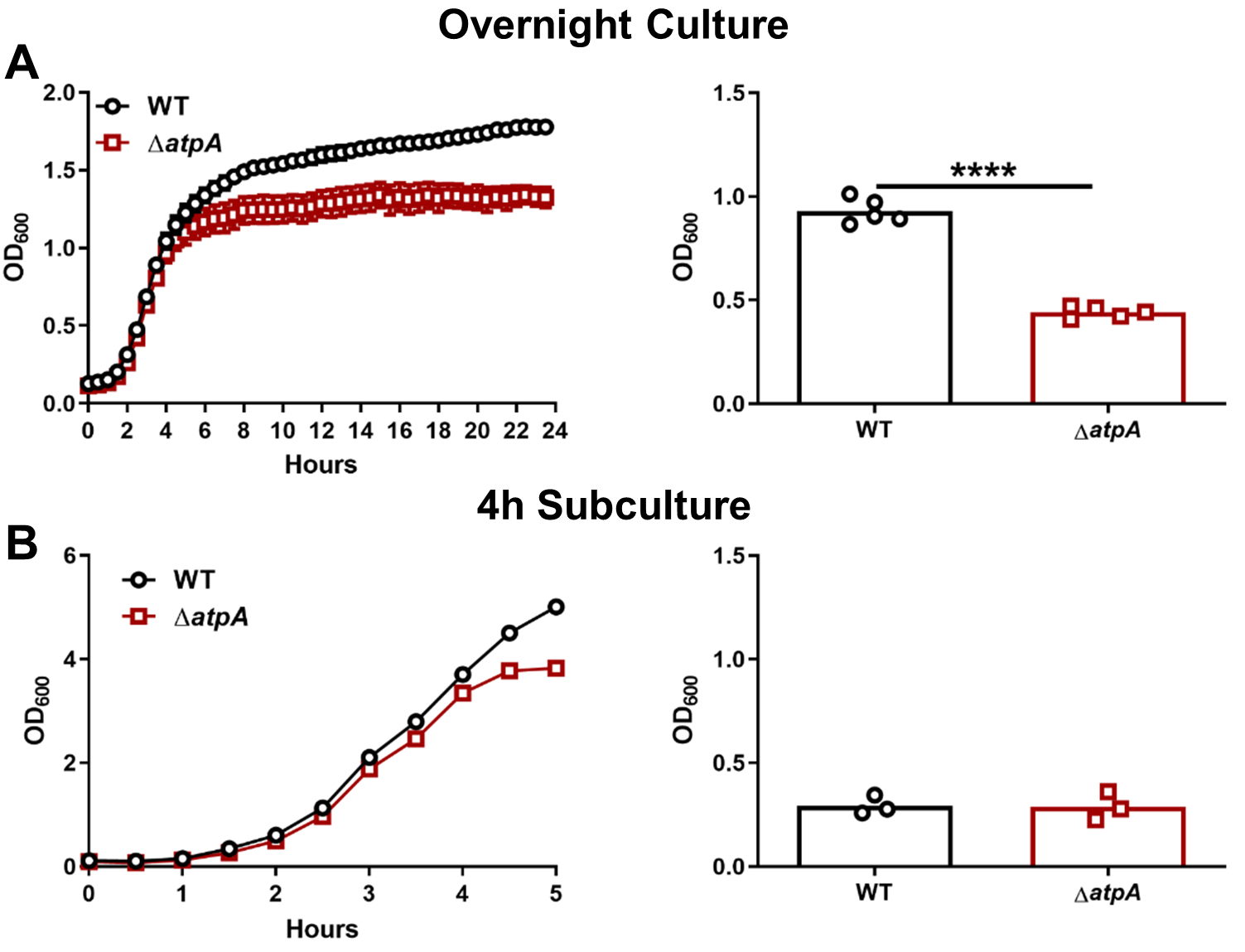

Supplement: FIG S5 [file mBio.01581-20-sf005.tif]

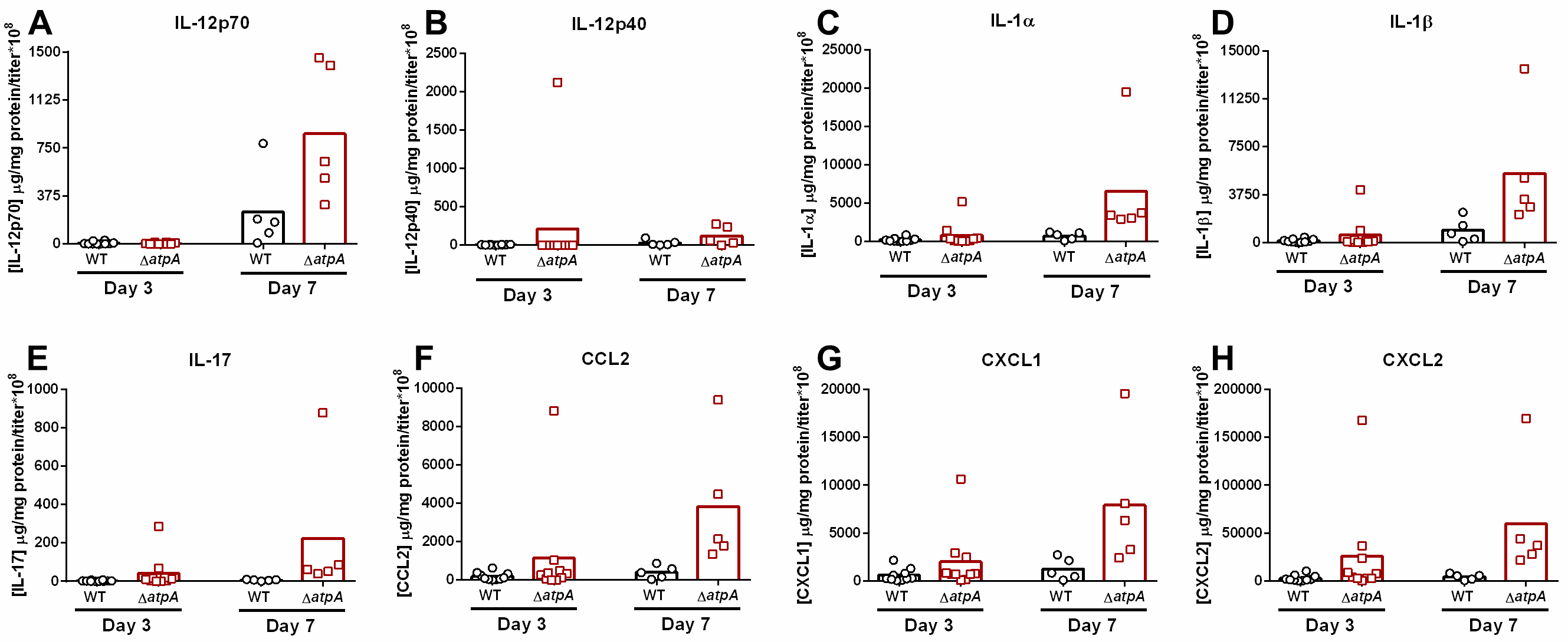

Supplement: FIG S6 [file mBio.01581-20-sf006.tif]

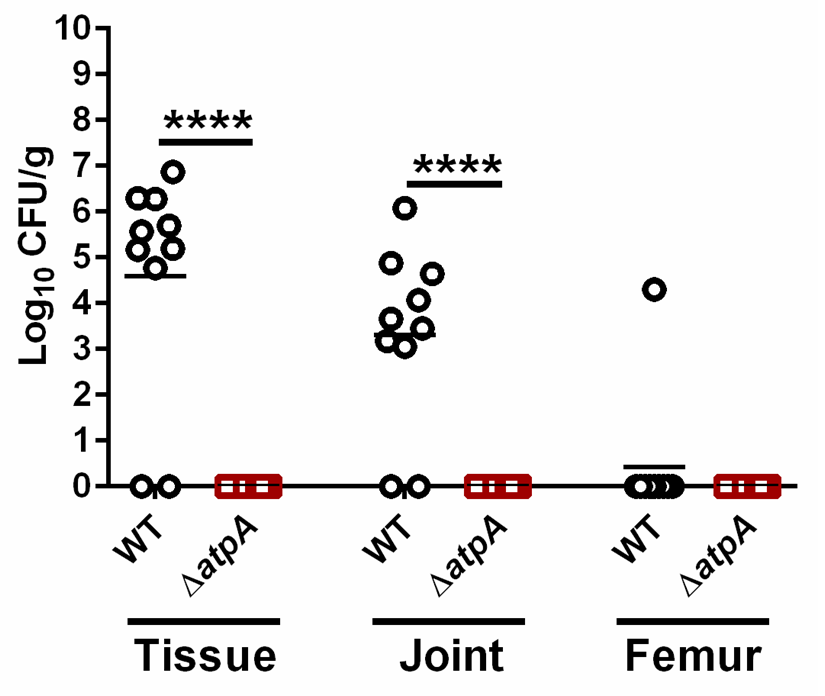

Supplement: FIG S7 [file mBio.01581-20-sf007.tif]
