# Supplementary material for: Staphylococcus aureus ATP Synthase Promotes Biofilm Persistence by Influencing Innate Immunity
Source: mBio. 2020 Sep 8;11(5):e01581-20. doi: 10.1128/mBio.01581-20 (PMC7482063; doi:10.1128/mBio.01581-20)
Supplement: TABLE S1 [file mBio.01581-20-st001.docx]

**Table S1. Bacterial strains and primers used in this study**

| **Bacterial Strain or plasmid** | **Relevant phenotype** | **Source** |
| --- | --- | --- |
| *S. aureus* LAC13c | Wild-type USA300 isolate | (50) |
| pCM29 | P*_sarA_*::gfp*_cmpR_* | (51) |
| Δ*atpA* | *S. aureus* LAC13c *atpA*::ΦΝΣ*_ermR_* | This study |
| Δ*atpA*::*atpA* | *S. aureus* LAC13c *atpA*::pAQ67 | This study |
| Δ*atl* | *S. aureus* LAC13c ∆*atl* | (50) |
| Δ*atpA*Δ*atl* | *S. aureus* LAC13c *atpA*::ΦΝΣ*_ermR_* ∆*atl* | This study |

| **Primer name** | **Sequence (5’-3’)** |
| --- | --- |
| pJB38_fwd | GTCGACCTGCAGGCATGC |
| pJB38_rev | TTGAAGACGAAAGGGCCTCG |
| ATPase_alpha_C_fwd | cgaggccctttcgtcttcaaAATTTAGAGACTATTAATGAAGAATTAAC |
| ATPase_alpha_C_rev | ttgcatgcctgcaggtcgacATAGCATGGTTAGCTAGTG |
| atpA_fwd | AGCTGAAGAAATCAGTGCATT |
| atpA_rev | CGTTAATTGCTGTGTCAAACTT |
| gyrA_fwd | GCCGTCAGTCTTACCTGCTC |
| gyrA_rev | AATAACGACACGCACACCAG |
| Atl_del_F | AGTTGCAGCAGCTTTAGAAGTAACTGATG |
| Atl_del_R | GGGATGACTTTTGATCCTATGTTCATGTTGC |
